# The Relation of Law to Social and Economic Conditions

**Published:** 1902-11

**Authors:** F. S. Key Smith

**Affiliations:** Rome, Ga.


ATLANTA

Journal-Record of Medicine.

Successor to Atlanta Medical and Surgical Journal, Established 1855, and Southern Medical Record, Established 1870.

Vol. IV. NOVEMBER, 1902. No. 8.

BERNARD WOLFF, M.D., EDITOR, Nos. 319–20 Prudential. Published Monthly. M. B. HUTCHINS, M.D., BUSINESS MANAGER, No. 64 Marietta St.

### *ORIGINAL COMMUNICATIONS.*

### THE RELATION OF LAW TO SOCIAL AND ECONOMIC CONDITIONS.*

By F. S. KEY SMITH, ESQ.,

Rome, Ga.

*Mr. President, Ladies and Gentlemen:*

It affords me great pleasure to be present at the first annual meeting of The Georgia State Sociological Society, and I esteem it a privilege and an honor to be requested, upon such an occasion, to read before you a paper. The society has my best and most sincere wishes for a long and successful career in the wide field and worthy task it has entered, and I feel sure it will accomplish much good. I believe the majority of your members are physicians or doctors of divinity. Not being of either of these estimable professions I must needs be a layman, as regards you. Nevertheless, your presence here indicates that you are interested in other

* Read before the Georgia Sociological Society, Atlanta, June, 1902.

matters than those strictly allied either with medicine or divinity; namely, the upbuilding of man and society, especially your country and state; for this reason I consider I am justified in speaking to you of the relation of the law to social and economic conditions, more as though addressing an assembly of members of my own profession. As I have delved into theories and conditions in the preparation of this paper, there has at times come to me suggestions that, while advancing in some respects, we were, in others, deteriorating as a nation. At other times my view would change, as though a panorama, and I could not restrain the conviction that such was impossible. To patriotic Americans I cannot conceive of anything which could cause more anxiety than even the intimation that our great commonwealth had reached its zenith and was starting down the slope towards its sunset horizon. Especially is this true of the young men of our country who, in the vigor of youth, with all before them, desire to take first place among the men of the world. To feel, therefore, that one's country is declining, in any respect, means that our field of action is diminishing and our opportunities growing daily less, which, to say the least, is not a pleasant thought to young men, aspiring to scale the dizziest heights, buoyant with the spirit of enterprise. As a young man let me ask, therefore, without wishing to be considered pessimistic, are there any here who, calmly viewing the events of the last ten years, feel perfectly secure in the future of our country? If not, the following observations will not be thought amiss to members of a society whose object is the improvement of the State. It has long been recognized of the law, that it is the bulwark of civil liberty—the protector of the rights of man and insurer of his peace and happiness. At all times, therefore, it is pertinent to inquire of its relation to present social and economic conditions. In the dealings of men, either as individual to individual, or in their aggregate capacity, as governments to individuals, or with other nations and foreign people, whether united in any recognized form of government or not, if the law is supreme, requiring all things to conform to its principles, justice flows, for the weak are protected against the strong, resulting in an era of general happiness and prosperity; but if conditions are allowed to shape the law, bending its principles so that they conform to the will of those in power, nothing is settled or certain, and a reign of terror and tyranny prevails, and that nation which would otherwise continue great, falls in ruins and decays, leaving only a struggling degeneracy as a reminder of past greatness. It is my present purpose, as far as possible, to demonstrate wherein we are deficient, for certain it is we are not perfect, and in what respects we are permitting other considerations than a love of justice and respect for the law to govern in our attitude towards our fellow-men.

Blackstone defines municipal law as "a rule of civil conduct, prescribed by the supreme power in a State, commanding what is right and prohibiting what is wrong." The first part of this definition alone is applicable to our present system—that is, "a rule of civil conduct, prescribed by the supreme power in a State," for the law does not always command what is right any more than it always prohibits what is wrong, unless it be contended that whatever the law commands is right and whatever it prohibits is wrong, irrespective of moral right and wrong. This, however, notwithstanding some have so claimed, I do not conceive was the meaning of the learned commentator. Without meaning to be egotistical, and with the proper deference for the able judge's opinion, I respectfully submit the following as a definition of American municipal law: "A rule for civil conduct, declared by those in authority, either legislative, executive or judicial, within the scope of their respective constitutional powers, expressing the public opinion of the age, sometimes free and just, but often influenced by the interests of wealth and then most frequently unjust. This is the law in the practical, unfettered by theory. If, therefore, practice be right, theory must be wrong and *vice versa*. Is it not time that mankind was deciding as between these, or are we to drift, rudderless, forever—some contending for theory, while others maintain and adopt the practice of the world? How often do we hear, "It may be all right in theory, but it will not work in practice!" This, if true, makes many beautiful theories and teachings all wrong. What is not possible of being carried out in practice has little, if any, support in fact, if merely right in theory. Acceptance of such a doctrine repudiates most of the teachings of Christ. Still we find many devout Christians in their daily intercourse with their fellow-men doing this, but who would become highly indignant were you to intimate anything of the kind. It is an easy matter to uphold the law and the gospel when speaking in the abstract, but quite another thing to do so when that law or gospel comes in conflict with our desires and interests; then it is we weaken, and seek a justification in the fallacious doctrine, that what is right in theory will not work in practice. We resemble the disappointed heir seeking, by any pretext, to break the will of the sire who has disinherited him. A member of the Catholic church once informed me that he was told by a priest at confessional, in reply to a question whether or not the usual business methods were justifiable in conscience, to do business as business is usually done. This able divine, while fully aware that many business methods are not reconcilable with the teachings of Christ, nevertheless recognized the fact that it is as useless for one man to stand out against the customs of the world, right or wrong, as it would be for him to stand upon the rock-bound coast of the Atlantic and endeavor to stem the ocean's tide. He, therefore, very diplomatically neither justified the ways of the world nor discouraged a member of his flock in his business career. But what a sad commentary upon man, who boasts that he is made in the image of God, possessed of a spiritual nature. Would it not be better were we, as a whole people, to accept the truth as our only rule of conduct as quickly in our daily practice as when reasoning purely in the abstract; and this, too, whether it runs amuck our desires and apparent interest, or is in harmony with them? What account is a doctrine that only holds good in theory? To follow truth for the sake of truth, believing in the long run our interests will be best subserved, is the key to the solution of most of the perplexing problems with which the world has to deal. Yet, with so simple a remedy at command, how many centuries of glowing temporary success, followed by permanent decay, both in men and nations, have passed and are yet to come, before men shall acknowledge the mandates of truth to be their only guide and rule of conduct? Then will history no longer repeat its sad stories, but a nation once great shall retain that greatness for all time, and generation after generation bequeath unto each succeeding one an era of still greater happiness.

The theory of the law is beautiful and, when properly administered, capable of performing justice in every instance. It is the best thought of the best thinkers among practical and experienced men uninfluenced by considerations other than a law of justice. In the administration lies all the difficulty. There do schemers strive with untiring energy to thwart its purpose, and to the shame of our system and nature must it be admitted, too often are they successful, frequently thus acquiring great riches, and with the power and influence which they bring discomfiting those who are less fortunate, because lacking the capacity and ability of their fellows, or, if possessing this, are not of the nature that can take the advantage which is, under our present methods, necessary to business success. As new conditions arise, it is true the law must grow, *not expand*, in order that they may be met. This is, however, essentially different from resorting to the truth of this principle as a means to evade the law—which has, of late, so often been done. Comparing our social and economic conditions with the above standard, we can discover whether the condition gives way to the law, or, according to the doctrine that what is right in theory does not work in practice, the law, although the true principle, is made to conform to the condition when the interests of wealth, combine and influence are involved. If the latter, much remains for the generations of this century to correct, that history may not with us repeat itself and we may be spared the same or a similar fate to the Roman empire and other nations of Europe. We are not justified in contenting ourselves with the thought, that what is will be and is right, for history will always repeat itself, notwithstanding our efforts. This cannot be if the conditions leading to any event are not the same or similar. It is wholly impossible, with the light of history to guide us; being in a position to control present conditions, if we will, we are in no small degree masters of the situation. True, there are many opposing forces, but in the ratio as they are overcome will our happiness and prosperity increase. I am speaking now as a whole people and not as a few individuals. Principal among them are worldliness, selfishness, an abnormal love of greed, and immorality. Eradicate these and we will have pretty nearly a perfect condition. Permit them to increase, and we shall have misery and decay in both people and state. A legal writer in one of his works, in speaking of worldliness, says: "It is true that a public and general extravagance in the ways of living would lead to national decay. Nations have often fallen into decay from the corruption caused by the individual indulgence of luxurious tastes." (Tiedm. L. L. P., 154.)

Whether we suffer from any, or all, of these principal evils must be determined by an investigation of our social and economic conditions and the relation which the law bears thereto. Considering first, trusts and large combinations, it requires little observation to discover that they are quite entirely actuated by selfishness and a love of greed. A certain amount of interest in finances is essential to the financial and commercial health of a nation, but when it exceeds the proper limit what before was food becomes poison. A short inquiry into the vigor and strictness of the common law in prohibiting and punishing love of greed will be of interest and beneficial by way of comparison with our law and conditions.

Says Blackstone. "The offense of forestalling the market is an offense against public trade. This, which (as well as the two following) is also an offense at common law, was described by statute 5 and 6 Edw. 6, ch. 14, to be the buying or contracting for any merchandise or victual coming in the way to market; or dissuading persons from bringing their goods or provisions there: any of which practices makes the market dearer to the fair trade. Regrating was described by the same statute to be the buying of corn or other dead victual in any market and selling it again in the same market, or within four miles of the place. For this also enhances the price of provisions, as every successive seller must have a successive profit. Engrossing was also described to be the getting into one's possession or buying up large quantities of corn or other dead victuals, with intent to sell them again. This must, of course, be injurious to the public, by putting it in the power of one or two rich men to raise the price of provisions at their own discretion. And so the total engrossing of any other commodity with an intent to sell it at an unreasonable price is an offense indictable and fineable at the common law (4 Bl. Com. 154; Tiedm. 242). When we consider the many large combines of our country, organized for the purpose of enhancing the price of commodities, one hardly realizes that such methods could ever have been the subject of criminal prosecutions, especially in the land from which we derive the better portion of our law. So too are we aghast at the disgraceful attempt of the present on the part of the large packing-houses to raise the price of meat, the effect of which is felt not alone in an advance in this commodity but in others as well. In fact, in a country boasting of its individual liberty and freedom alike for all, there are many families to-day who cannot afford to eat meat. Is it to this our glorious American commonwealth has come? A land stained crimson by our martyred forefathers for the sake of justice —an aristocracy of wealth, the most to be feared of any the world could know; no iron rule of kings will ever equal it in tyranny and oppression, if once it is yoked upon us.

Mammon, the god to be worshiped—truth and justice, satans to be shunned. Surely the time has arrived when the American people should awaken to their economic conditions, and nothing demands their attention more at present than the proper regulation of trusts and corporations. While we dispute over conditions in far distant islands our own country is growing up in weeds, filled with venomous reptiles of every kind and description, striking and hissing at each other, which before long must mean death to the nation. These trusts and combinations rarely stop at anything when determined on carrying their point. An instance occurs to me just now. About a year ago when oil was creating a fever in Texas, I was informed that the Standard Oil Company, failing in an indeavor to purchase certain tracts and wells, which would have given them a controlling interest, resorted to an expedient to force a sale; buying up a great portion of wharf property in Galveston and elsewhere, over which it was necessary to ship the larger part of the oil from these wells.

With the control of the channels of exportation the advantage thus acquired can be readily imagined. Were I upon the bench and a case of this kind came before me, I should have no hesitancy in setting aside every deed as being contrary to public policy, and in doing so could find ample authority in the common law of the land to support me. Why, then, are such frauds permitted? Have we not legislatures and courts for the making and enforcing of the law? The reason is this, our great commercial success in the past century has warped the minds of us all, legislatures and judges alike. In granting license and liberty to the speculator and manufacturer, we have overstepped the bounds of propriety to the great detriment of the masses. Hear what a writer says: "A man has a constitutional right to buy anything in quantity, providing he use only fair means, and set his own price on it, or refuse to sell at all. Where one man, acting independently, does this, he can be only considered guilty of a wrong to the public, when he secures the possession of these things by the practice of fraud, or endeavors by false reports to enhance the price of a commodity which he offers for sale. . . . . Further the law cannot go." (Tiedm. 244.)

Mr. Bishop, in discussing the common law offenses of regrating, forestalling and engrossing, says: "In reason, the essence of the common law on the subject of forestalling, considered distinct from engrossing and regrating, seems to be that, whenever a man, *by false means, or by any kind of deception*, gets into his hands a considerable amount of any one article of merchandise, and holds it for an undue profit, thereby creating a perturbation in what pertains to the public interest, he is guilty of the offense of forestalling." "As stated by Mr. Bishop, the common law," says Mr. Tiedman, "in making a criminal offense of forestalling, is no more open to constitutional objection than the punishment or prohibition of any other act of fraud or deception." But Mr. Bishop's position, in regard to engrossing, is not as free from criticism. He says, whenever a man, for the purpose of putting things, as it were, out of joint, and obtaining an undue profit, purchases large quantities of an article of merchandise, to hold it, not for a fair rise, but to compel buyers to pay a price greatly above, as he knows what can be regularly sustained in the market, he may, on principle, be deemed, with us, to be guilty of the common law offense of engrossing." "It is without doubt an immoral act," continues Mr. Tiedman, "to ask an unconscionably high price for a commodity, taking advantage of the pressing wants of the people; and it may, under a high code of morals, be held to be an extortion for one to purchase and hold merchandise for the purpose of gaining from its sale more than a fair profit; but it cannot be claimed that there is a trespass upon the rights of others in doing so, or that the rights of others are thereby threatened with injury." (Tiedm. p. 244.) With these conclusions I cannot agree as to me. This wholesale buying up of commodities by the rich few for the purpose of extorting a larger price from the poor, their less fortunate fellows, who compose by far the greatest bulk of society, must necessarily work an injustice and hardship upon the great mass of the people. I cannot see, therefore, why under the principle that no one can use his own in a way prejudicial to the rights of others, it may not be prohibited whether fraud is used or not; and for a learned author to argue differently only illustrates how the mind can become warped by long acquiescence in evil customs, another care which should be exercised that they be not established. If such buying up and selling by the individual be wrong, what is the case with conspiracies between men and combinations of men, for the purpose of using their combined efforts to oppress the weak and poor. With such conditions existing have we not passed the mark where healthy interest in enterprise ceases and an undesirable love of greed begins? Besides are we prepared to say that fraud does not enter into such transactions?

As expressed by one judge, "A combination is criminal, whenever the act to be done has a necessary tendency to prejudice the public; or to oppress individuals." (Commonwealth *v.* Carlisle, Brightley 40.) Are not the trusts doing this? Why then are they not properly controlled? We find the answer in the following extract from Mr. Tiedman's work on the Limitation of Police Power. He says: "But the illegality of combinations is pushed to the extreme limit when it is held that a combination to enhance the price of a commodity is always unlawful even where there is no deception or fraud, and when the combination do nothing more than hold the goods which they control for higher prices. *But that is the common law rule. Such combinations are quite common in later days, and public opinion is very tolerant of them, rarely, if ever, condemn-*

*ing the practice as immoral, but there can be no question concerning their illegality."* (Tiedm. 249.) Furthermore, Mr Tiedman observes, "since the common law made it an indictable offense for one man to corner the market, there can be no question that the combination of two or more to buy up any article of merchandise, and force the payment of exorbitant prices, is a criminal conspiracy, and may be punishable without further legislation, *if public opinion did not look so leniently upon such transactions."* (Tiedm. 251.) The fault in this, however, lies in the fact that public opinion is rarely measured by the opinions of the masses, but by that of the comparative few who influence the affairs of the world. If, however, public opinion is so thoroughly debased, we need to create a more healthy public sentiment. There exists a strange anomaly in our law in these cases. This precludes the recovery of money paid under these agreements and combinations as being unlawful, yet permits these large combines to exist and operate. The courts, by an unbroken trend of authority, hold, and in most instances independently of statute, that money advanced for the purpose of cornering the market cannot be recovered by any legal measure; that all such agreements cannot be enforced in the courts, the law leaving the parties, as it expresses it, where it finds them. But this is the limit of the authority of the courts, without legislation. In the light of these decisions what is the matter with our legislators? Why is not the power to prosecute such illegal schemes and conspiracies confined upon the courts and the proper officers? And why, when, as in a few instances, some, though inadequate, legislation has been enacted, are there not more prosecutions and convictions? Because, as a judge in Michigan has said, "There is no doubt that modern ideas of trade have practically abrogated some common law doctrines which are supposed to unduly hamper commerce . . . . But we do not feel called upon to regard so much of the common law to be obsolete as treats these combinations as unlawful, whether they should now be held punishable as crimes or not." (Raymond *v.* Leavitt, 46 Mich. 447.) As expressed by another court, "There may be some difficulty in determining such conduct to be in violation of public policy, where it has not before been covered by statutes as precedents. But in the case before us the conduct of the parties comes within the undisputed censure of the laws of the land, and we cannot sustain the transaction without doing so on the ground that such dealings are so manifestly sanctioned by usage and public approval, that it would be absurd to suppose the Legislature, if attention were called to them, would not legalize them. We do not think public opinion has become so thoroughly demoralized; and until the law is changed, we shall decline enforcing such contracts. If parties see fit to invest money in such ventures, they must get it back by other than legal measures." (People *v.* Fisher, 14 Wend. 9.) I have quoted thus at length from the opinions of the courts, that it may be seen the fault lies not with the judiciary but with the legislature, where the opinions and influence of men of affairs, the interested parties, masquerading as public opinion when the masses cry out beneath the burden, unheeded, for mercy, have their weight.

With one other quotation in this connection I shall pass from the subject of trusts to another closely allied therewith and for whose existence large combinations of capital are chiefly, if not entirely, responsible. Says Mr. Tiedman: "Of the same character would be an agreement between all the transportation companies of a particular territory, which was made for the purpose of preventing competition and controlling the rates of charges for transportation. Such agreements are void." (Mr. Tiedman means by this, the courts pronounce and hold them so when they come before them in suits between the parties thereto for enforcement.) "The only ground," continues Mr. Tiedman, "upon which the prohibition of combinations in such cases may be justified is that such combinations tend to give to the members of them an undue and dangerous power over the needs and necessities of the people, and for that reason it is a legitimate exercise of police power to prohibit such combinations. Such a law does not interfere with the equal freedom of all to do what they will with their own. Every one is left to do or act as he pleases, but he is not allowed to deny to others an equal freedom, not even with their consent. Public policy, the public safety, requires the prohibition." (Tiedm. 251.) Yet with the law thus plainly stated, how many prosecutions and convictions take place yearly, when daily all over the country the law is being openly violated.

In 1886 it was said: "The sharp competition of modern trade; the rapid increase in the productivity of labor-saving machines—in fact, all the characteristics of modern industry tend to sharpen the struggle for existence on the part of the weaker; and the latter has presented to him the alternative of barely eking out an existence on a mere pittance, or wresting by unlawful means a more comfortable living from those who, by a superior physical or intellectual strength, or by chicanery, have been able to gather together an undue share of the world's wealth, and public disorder and general insecurity ensues." (Tiedm. 254.)

If these conditions existed in 1886, how much have they increased in 1902? With such facts before us, is it not to the interest, as well as the duty, of the more intellectual and influential to adopt some measure or measures looking to the alleviation of the wrongs and sufferings of the less fortunate who compose by far the greater part of society? For, as Abraham Lincoln once said, "God must have loved the common people as he made so many of them." That it is to our interest and duty to do so admits of no doubt, and but for the love of the world, greed and self me might have hopes. Let us hope anyhow, and may this society be a mover to better things and conditions. Have we any right to wonder at the laboring classes, suffering and chafing under the injustice done them by large combinations of capital, organizing in order to acquire the strength to protect and maintain their rights? Is it not in compliance with the first law of nature—self-preservation? If the more intelligent and powerful will not, from a broad view of their own interest, protect the weak, but persevere in combining and conspiring against them, should they marvel at the unfortunate ones exerting every effort they may devise to better their condition? If so, why not question their own desires to do the same thing? Of course, I do not uphold or sanction the extremes to which labor organizations often resort, nor the bloodshed incident to their strikes and riots. But let me ask this question? Where in truth does the blame belong? With the laborers or with the more intelligent, who pass wakeful nights devising schemes which oppress, and at the laborer's expense to put more money in their already overflowing pockets? I do not advocate protecting the laborer in his illegal acts any more than I do the capitalist and trust magnate. But I do say both should be dealt with equally and fairly. Sir James Fitz-James Stephens, in the third volume of his history upon the criminal law, says: "At common law," which means the law independently of statute as adjudged by the courts, "no person was ever convicted for having combined with others for the raising of wages." This would make such conspiracies or combines, if criminal at all, statutory offenses. That we may the better understand this subject, let us briefly sketch the history of trade unions as shown by the early legislation in England affecting them. We have to some extent reviewed the English legislation upon the offenses of forestalling, engrossing and regrating, prosecutions for which lasted until 1844, when these offenses were abolished in England by statute 7 and 8 Victoria, ch. 24. The first legislation relating to trade unions was the statutes of laborers, passed in 1349 and 1350, being the 23 Edward 3 and 25 Edward 3. These statutes compelled all laborers to work when called upon unless they had sufficient means of support, and fixed the wages of the more important classes of mechanics. By statute 3 Henry 6, passed in 1424, it was enacted that, "Whereas, by the yearly congregations and confederacies made by the masons in their general chapters and assemblies, the good cause and effect of the statutes of laborers be openly violated and broken, the chapters should not be holden; those that caused them to be assembled and holden should be judged for felons, and all masons coming to such congregations should be punished by imprisonment, fines and ransom." (Stephens's Hist. Cr. L. 205.) In 1548 a more general statute was passed, the 2 and 3 Edward 6, which prohibited all conspiracies and covenants of laborers, artificers, etc., for the purpose not to labor except for a certain price or rate. An act of 40 George 3, passed in 1800, remained in force nearly twenty-five years, which, in a word, prohibited and punished all combinations that with us to-day are trade unions, and each and every act which they are accustomed to do in endeavoring to enforce their demands. The sweeping severity of this is not surprising under George the Third. Sir James Fitz-James Stephens, in speaking of these statutes, says:

"I should not myself describe it as a system specially adapted and designed to protect freedom of trade. The only freedom for which it seems to me to have been specially solicitous is the freedom of employers from coercion by their men." (3 Vol. History Cr. L. p. 209.) So stood the law up to 1824 and 1825, when it was changed and placed upon an entirely different plane, permitting what had before been unlawful so long as violence was not done to either person or property, or force used in preventing others who might desire to work for the wages given from doing so. However, the innovation made by these statutes was greatly abridged by the decisions of the courts, and it became necessary in 1859 for Parliament to pass the 22 Victoria, ch. 34, which provided, "That workmen were not to be considered guilty of 'molestation or obstruction' (two terms used in prior statutes,) under the act of 1825, by reason only of entering into agreements for the purpose of fixing the rate of wages, or hours of labor, or of endeavoring peaceably to persuade others to cease or abstain from work in order to produce the same effect." (Stephens's History Cr. L. p. 221.) Up to 1883 an act passed by Parliament in 1875, entitled The Conspiracy and Protection Act, 38 and 39 Victoria, ch. 86, was the last English legislation upon the subject. This act provided first, that "an agreement or combination by two or more persons to do or procure to be done any act in contemplation or furtherance of a trade dispute between employers and workmen shall not be indictable as a conspiracy, if such act committed by one persen would not be punishable as a crime." (3 Stephens's History Cr. L. 225.)

Mr. Bishop states that in England and this country, combinations among workmen to raise the price of wages are indictable at common law, but from the above it will be seen that such is not the case, and that these offenses are purely statutory. Besides in England, since 1825, the severity of these statutes has been very much mitigated. It may here be remarked that so long as forestalling and engrossing, whether by the individual or by combinations, were made indictable, thus protecting the laborer against the wealthy and combinations of capital, there may not have been any great hardship or injustice in prohibiting organizations of labor, especially when the law fixed the hours for work and the rate of wages. But in the state of our law and conditions, where the offenses of forestalling, engrossing, etc., are unknown, and immense combinations of capital are the order of the day, without any regulation of wages by law, to permit the stronger to reduce the rate of wages and raise the price of necessary commodities, and then prohibit and punish the laborer for organizing to protect himself, is an injustice which God will not long tolerate, even in his much-favored American people. What the intelligent do ,by chicanery, the laborer knows only how to accomplish by force, and while neither is right, force in one is not worse than chicanery and trickery in the other; therefore, neither can be justified and neither should be pardoned. Labor organizations, so long as they have for their object the protection, by fair means, of the laborer in his right to receive a reasonable compensation for his labor as compared with the profit made on the sale of any commodity which he produces, are not unlawful, as we have seen is recognized by the latest English statutes. Especially is this true when such organizations are caused by the oppressions of employers. To justify remedial legislation of the weaker, the stronger should receive it first. No doubt both should be regulated, but to regulate one without the other is unlawful and unjust.

It may here be asked what remedy would I propose. To suggest a remedy it must be admitted is always much harder than to point out the evil to be remedied. Still, I am not without ideas upon this phase of the situation, which briefly are as follows:

I should, in the event these rapidly increasing combinations of capital and labor cannot be checked or *controlled* satisfactorily in any other way, give the management of every large combine, trust, or corporation into the hands of the government to be operated in a similar manner as is the post-office department at present. I am aware that this may not at first meet the approval of all, but believe as the question is studied it will appear, certainly in the event of no other satisfactory solution, to be a plausible, if not the only one. The objection that this will give the government too much power I do not think well founded, any more than the management of the post-office has had this effect; and so long as the people have a voice in the election of the public officers who would, under such a system, operate these immense commercial industries, they would be in a position to exert, at least, some power and control over the industries themselves. As it is at present they have none; neither are the men operating these enterprises responsible to any save themelves. It may also be asked: In this event what would you do with the labor organizations? I answer, control the trusts, and labor will regulate itself. No man will rebel against the rates paid by the government for his services, especially when he has a voice in the fixing thereof. It has alone been the question of wages which has given birth and nourishes organized labor. Once protected in his compensation, the laborer will cause little annoyance, and their organizations become quite inactive, if they do not cease to exist.

Besides, having regulated the trusts, it would be lawful and just to pass suitable legislation for controlling labor, if needs be. Another inquiry that suggests itself to the mind of an inquiring student of our social and economic conditions relates to the morals of our people. For my part, I regret exceedingly that I am not favorably impressed. I appreciate, however, with those who lead quiet and retiring lives, spending the greater part of their spare time at home, seldom frequenting cafés, hotels, theaters, and other places of amusement, their experiences may cause them to differ with me. Nevertheless, I feel reasonably safe in asserting that those who have mingled or mixed with the world to any extent have seen many things which would put our colonial ancestors to the blush. In a word, I regret I feel, from what I have heard and read, we have degenerated in morals, as we have grown as a nation, within the past fifty or seventy-five years. It was not longer than six weeks ago, when sitting with a friend one evening in a fashionable café of Washington City, that I was somewhat amazed at the number of society women who came in while we were there with their escorts and partook of whiskey high-balls and various other drinks, beer being the most seldom called for.

In commenting upon this, I observed that I thought we were, as a people, becoming very lax in our morals, to which my friend replied. No! Not for a nation of our wealth and standing, for we are the most moral the world has ever known. I am glad to be able to say, when viewed from this standpoint, I can agree with him. Nevertheless, when taken in the light of a comparison with the morals of our ancestors of fifty or seventy-five years back, I fear, from all I can learn, we have in this respect degenerated. Besides, in considering this question in the light of a comparison with other great nations, regard should be had for our youth. If the acquisition of wealth and greatness is concomitant with a pro rata decline in morality, there is much that remains to be done in guarding against this evil, and we should lose no time in elevating ourselves to a higher plain. No law can accomplish this. Our redemption lies in soaring into a higher atmosphere. On the other hand, not wishing to be misunderstood, I will add, I am not among those who consider it a sin for a man to take a drink, provided he knows where, when, and how to do so. I consider it entirely different with a woman, however, especially in public places, and regret it is becoming fashionable.

Time precludes my dwelling longer upon this subject, nor is it necessary. Every one is free to draw his own conclusions from his personal observations. I may add, however, as a general rule, where there is great extravagance, immorality nearly always exists. That we are living in an extravagant age I hardly think will be denied. One of the most positive evidences of our moral degeneracy is the rapid increase in divorce and the light regard in which the sacred obligation of marriage is held. If there is anything upon which we need a uniform system of law it is, marriage and divorce. It may almost be wondered why the framers of the Constitution did not give Congress in this, as in bankruptcy and other things, authority to establish a uniform system for the United States. This, however, does not appear so remarkable when it is remembered how little need there was for divorce laws in those days, as divorces, when obtained at all, were granted by the colonial legislatures, which of itself would indicate our degeneracy in morals when compared with our American ancestors. On the other hand, in dealing with this most important subject at the present time, care should be taken that we do not allow too high a regard for morality to carry us away. The grounds for divorce should not be confined to adultery, for in their practical effects upon the wife and family habitual drunkenness, extreme cruelty and desertion are as equally injurious, and the wife and children should be protected against all. Closely allied with this subject, although of a different nature, are the comparatively few marriages of the present day and number of estimable young girls and women who are obliged to do a man's work in order to make a support, not only unsexing them, but unfitting them for the duties of domestic life, while the men squander their earnings rolling in high life, as it is termed. Argue as you may, such conditions are against the nature of things, and must in the end bring despair to the nation and its people wherein they exist.

The relation of the law to the sale of alcohol is of the utmost importance and interest. Mr. Tiedman, in speaking of the laws prohibiting the sale and manufacture of liquor, observes: "It is no wonder, when the zealous reformer contemplates the care-worn face of the drunkard's wife and the rags of his children, that he appeals to the lawmaking power to enact any and all laws which seem to promise the banishment of drunkenness; forgetting, as it is very natural for him to do, since zealots are rarely possessed of a philosophical and judicial mind, that to make a living law, it must be demanded, and its enactment compelled, by an irresistible public opinion; and where the law in question does not have for its object the prevention or punishment of a trespass upon rights, it is impossible to obtain for it the enthusiastic and practically unanimous support which is necessary to secure a proper enforcement of it. Furthermore, if in any community public opinion is so aroused into activity as to be able to secure the enforcement" (not the passage bear in mind) "of a law having for its object the prevention of a vice, the moral force of such a public opinion will be amply sufficient to suppress it." (Tiedm. 299.) "The object of police power is the prevention of crime, the protection of rights against the assaults of others. The police power of the government can not be brought into operation for the purpose of exacting obedience to the rules of morality, and banishing vice and sin from the world." (Tiedm. 150.) Besides the rule of law is, that "no trade can be subjected to police regulation of any kind unless its prosecution involves some harm or injury to the public or third persons, and in any case the regulation cannot extend beyond the evil which is to be restrained . . . . No trade can be prohibited altogether, unless the evil is *inherent in the character of the trade*, so that the trade, however conducted and whatever may be the character of the person engaged in it, must *necessarily* produce injury upon the public or upon individual third persons." (Tiedm. 301.) With these quotations on the law it is interesting to compare the methods adopted in some places in prohibiting liquor selling. This trade is not necessarily injurious, in a legal sense, to the public ; and where injury does result, it is generally from the weakness or wrong of the consumer, which wrong is not participated in by the seller when he does not know, or cannot be supposed to know, that intoxication is likely to follow. Thus it is demonstrated that a law prohibiting absolutely the sale of liquor is unconstitutional, as we have seen a trade cannot be prohibited altogether unless its prosecution is necessarily injurious. The passage of such a law, therefore, is in violation of the fundamental law of the land and the rights of the people, and upon the principle that two wrongs do not make a right can not be justified. Such a law is what we term a bad law. It is bad because it abridges the rights of the people. Secondly, because it is very hard, if not impossible, to obtain convictions for its violation. Thirdly, experience has shown that such laws are always violated and frequently quite openly. Not very long ago I heard a prominent citizen and ex-member of the legislature of this State admit, when he needed whisky for medicinal and household purposes, living in a county where there was local option, he would give a negro a dollar and the negro in fifteen or twenty minutes would bring him the whisky.

I asked him where the negro got the forbidden fruit and was told that there were plenty of "blind tigers," or "speak easies."

This same gentleman was an ardent advocate of prohibition and helped in the passage of the law which gave to his county local option. I asked him if he did not regard this violation of the law in which he, the negro and the keeper of the "blind tiger" participated a greater evil than the trade, it necessarily tending to weaken reverence for all law, consequently diminishing its effectiveness as a restraint upon wrong and crime. To this he made no reply. There can be no doubt that in a small town a large number of common barrooms, little above the order of dives, situated upon the most prominent streets, near schools, churches and shops, where our wives, children, mothers and sisters have frequently to pass, are hardly to be tolerated, and we cannot wonder at the just indignation of the better class of inhabitants, nor their exerting every effort to suppress and abolish such nuisances. Besides, there is nothing illegal in their doing so, provided they proceed in a lawful manner and do not allow their ardor and enthusiasm to run away with their wisdom and sense of justice. Frequently, however, the fault is largely with the town authorities if their saloons are of a low order and situated in places and localities where they become nuisances. A proper town ordinance, strictly enforced, regulating the hours of opening and closing and the localities where saloons shall be kept, would have all the beneficial effect desired without abridging the personal or property rights of their owners; while caution and care in issuing license to only such men as might be relied upon and who could furnish bond for the proper conduct of their places would rid the town and community of the low grog-shop. With a few observations upon the dispensary method of dealing with the liquor question I shall pass to my concluding topic. I cannot, with truth, say that I favor even this prohibition, in a limited way. From all I gather, the scheme has been tried for some time in South Carolina but has not met with any phenomenal success or approval; while, on the other hand, it has been the cause of much scandel and vituperation against those who have controlled and operated the dispensary.

So far as diminishing drunkenness, I doubt, from the very nature of the thing and a knowledge of human kind, whether it does so. True, it may prevent the moderate drinker from taking many a glass that he would otherwise could he drop into a saloon and get one. But these do not compose the class of drinkers who menace society. The hard drinker, or inebriate, will get his whisky just the same, whether he has to buy it by the drink or by the bottle, which results in his spending fifty cents where before he spent a dime, and his taking his grog by the pint instead of by the gill. This is my candid and unbiased judgement upon this most important and serious question. I have always thought, and still think, that a high license cautiously issued, together with proper town ordinances regulating the localities where intoxicating liquors may be sold, the hours for opening and closing—with prompt arrests and fines imposed upon both the intoxicated and the saloon-keeper who sells to one intoxicated, or whom he had reasonable grounds to know or believe would become so, is the only proper and effective method of dealing with this question. Furthermore, when the law attempts more it goes outside its pale. As Mr. Tiedman observes: "The people at large cannot be made to feel sufficiently acutely the necessity of enforcing these laws, in order to make them effective remedies for the suppression of the evil. A man sees a pickpocket steal his neighbor's purse while on his way through the public streets. He will instantly, and involuntarily, give the alarm and render whatever aid possible with him in securing the arrest of the offender. The same man, a few steps further, sees another violating the law against the sale of intoxicating liquor, and although he may be an active member of some temperance organization, he will be sure to pass on his way, and say and do nothing to bring this offender to justice. Why this difference of action in the two cases? In the first case, the act was a trespass upon the right of property of another, and self-interest, through fear of a like trespass upon his own rights of property, prompted the man who saw the crime to aid in the arrest of the criminal. In the latter case, no man's rights were trampled upon; the unlawful act inflicted no direct damage upon the man who witnessed the violation of the law, and consequently self-interest did not impel him to activity in support of the law." (Tiedm. 300.)

In concluding permit me touch upon a subject in which all nations, to the present, have been and, although slowly improving, are still much negligent: the treatment and control of the criminal class, who are to both personal and property rights the greatest menace. Were we to expend one half the study and time in discovering methods for the treatment of criminals that we give to other subjects of far less importance, we would within a century reduce crime and criminals from what they are at present by quite three fourths. As a paper by me upon this subject was read before the society in December last, I shall not now attempt more than a brief statement of my views upon this interesting question, prefacing them with a few conclusions from a paper which was recently sent me by its author. In this he states:

"The following statements as to the criminal are not based upon experimental research so much as upon the experience of those who have studied criminals directly, or who have had practical control of large numbers in prisons or reformatories:

1. The prison shall be a reformatory and the reformatory a school. The principal object of both should be to teach good mental, moral, and physical habits. Both should be distinctly *educational.*

2. It is detrimental financially, as well as socially and morally, to release prisoners when there is probability of their returning to crime; for in this case the convict is much less expensive than the ex-convict.

3. The determinate sentence permits many prisoners to be released who are morally certain to return to crime. The indeterminate sentence is the best method of affording the prisoner an opportunity to reform without exposing society to unnecessary dangers.

4. The ground for the imprisonment of the criminal is, first of all, *because he is dangerous to society.* This principle avoids the uncertainty that may rest upon the decision as to the degree of freedom of will; for upon this last principle some of the most brutal crimes would receive a light punishment. If a tiger is in the street, the main question is not the degree of his freedom of will or guilt. Every man who is dangerous to property or life, whether insane, criminal, or feeble-minded, should be confined, but not necessarily punished.

5. The publication in the newspapers of criminal details and photographs is a positive evil to society, on account of the law of imitation; and, in addition, it makes the criminal proud of his record, and develops the morbid curiosity of the people; and it is especially the mentally and morally weak who are affected.

6. It is admitted by some of the most intelligent criminals, and by prison officers in general, that the criminal is a fool; for he is opposing himself to the best, the largest, and the strongest portion of society, and is almost sure to fail."

I also quite recently read an article by an English writer, Sir Robert Anderson, whose ideas to some extent coincide with mine. He advocates an inquiry into the past life and antecedents of every convicted fellow before sentence, and then, as it is established whether he is an occasional or a habitual criminal, a sentence to fit the criminal and not the crime. Such a course would certainly be a step in the right direction, but the greatest objection to it is the expense and time incident to such an investigation, and the opportunity afforded for fraud and perjury, to say nothing of the inability of the courts to conduct, with any degree of satisfaction, such an investigation. I am, therefore, still of the opinion that the best method is confinement in an asylum, presided over by a responsible board of able criminalogists, for an indefinite period, where the inmates can be watched and studied. The worst eventually being separated from the better and each treated accordingly; all being taught that release from their confinement rests entirely with themselves by a positive demonstration of their ability and determination to lead proper lives if returned to society (which might at first be on parole) and given an equal chance to earn an honest living. As I have endeavored before to show, such a course would rid society of every habitual and incurable criminal, as being thus confined, not permitted to marry to perpetuate his class, he would soon pass away. To a certain extent he would be a charge upon the State, but one which would decrease in proportion as his kind died. The better element, I believe, would improve and gradually be returned to society, where they would, in more than a reasonable probability, lead decent and useful lives. Such a system is now in operation, I have very recently discovered, in the Reformatory at Elmira, New York, and works admirably. Here they take in offenders up to thirty years of age—the idea seeming to be that one having followed a life of crime for thirty years and upwards is too steeped in evil ways to be capable of reforming. All of which may be true, but how about the case of one who does not commit his first crime until after he is thirty years of age? Should he go to the penitentiary to further learn evil ways or to a reformatory to repent and reform? Also, how about the protection to the State afforded by the indefinite confinement of habitual and incurable criminals? Nevertheless, the Elmira Reformatory is doing splendid work, and I trust the time is not far off when we shall have such institutions in every State of the Union, only without the age limit. Be this as it may, it must be admitted that the relation of our present system of criminal jurisprudence to crime and criminals is far from adequate, and does not accomplish the ends for which it is instituted. As enlightened American citizens of the twentieth century, of which we are so proud to boast, is it not to our interest, from a business standpoint as well as a moral duty, to give a graver and deeper study to this most important subject? It will not do to say that so long as I never expect to commit a violation of the law of my country, much less be guilty of the commission of a crime, I am not personally interested in those who do, or the punishment which the law metes out to them. As crime and criminals attack both the personal and property rights of every citizen, the latter has a personal interest of the deepest kind in this question from a selfish and business standpoint, to say nothing of that created by his moral and patriotic duty. Let us hope for better things. The minds that have solved the great economic and political problems of ages can as readily find a solution for this if their attention is given with sufficient enthusiasm and strength of purpose. After all, I believe if a solution be once undertaken in the proper spirit it will be found that crime is one of the easiest problems with which we have to deal. The whole trouble in the past and at present is the lack of proper interest and attention. Too much revenge enters into our methods. It is true "revenge is sweet"; but it is likewise true, God saith, "vengeance is mine."

I shall be more than repaid if I have interested you and have not been tiresome. I have regretted somewhat the length of this paper, but it was impossible to make it shorter and do justice to my subject. I fully appreciate the magnitude of the questions with which I have attempted to deal and in the preparation of this far from competent paper. I trust my effort has not been in vain, although my task has been none of the easiest. If what I may have said be worthy of lodgment in the hearts of others more capable of remedying existing evils, I shall indeed be happy. From what I have said, however, I do not wish to be thought either an alarmist or a pessimist. I could as readily write as much and as ardently upon the many good things in our people and nation—the finest the world has ever known, but there are certain evils, above enumerated, eating at the root of the good fruit, which I desire to see destroyed, that the good may live and the better thrive. Enjoying youth and health, the blessings of love of wife and child and citizenship in this great commonwealth, what reason is there to be other than happy? But an enthusiastic nature, with an ardent love for the pure success of one's country and the most perfect happiness of its people, chafes when it sees things "out of joint," and the divine principles of justice and truth, which nature has implanted in the breast of every man, made to give way to conditions, *when* conditions should bend and conform to them. Never has there been a greater truth stated than when Shakespeare made Portia say to Bassanio in reply to this appeal:

> "Wrest once the law to your authority;
> To do a great right, do a little wrong;
> And curb this cruel devil of his will."

Her answer:

> "It must not be; there is no power in Venice
> Can alter a decree established;
> 'Twill be recorded for a precedent;
> And many an error, by the same example,
> Will rush into the State. *It cannot be.*"
>
> —*Merchant of Venice.*

Could we have more judges and public servants with this strength of character, the law, carried out in practice as it is found in theory, would secure to all the reward due thought and deed, be they the rich or poor, the influential or the weak; and talent and honesty of purpose would reign ahead of wealth and shrewdness.

Thanking you for your kind attention to my humble remarks, in the words of an American poet:

> "Let us, then, be up and doing,
> With a heart for any fate,
> Still achieving, still pursuing,
> Learn to labor and to wait."

I bid you good-night.

